# Selection of a Potting Material and Method for Broadband Underwater Cymbal Arrays

**DOI:** 10.3390/s22218324

**Published:** 2022-10-30

**Authors:** Wenbo Wang, Hayeong Shim, Yongrae Roh

**Affiliations:** School of Mechanical Engineering, Kyungpook National University, Daegu 41566, Korea

**Keywords:** cymbal array, potting material, potting methods, finite element method

## Abstract

Cymbal transducers are often used in arrays for underwater communication and detection systems. The working environment of a cymbal array is underwater; therefore, waterproofing, salt-corrosion prevention, and impact resistance are necessary for stable operation of the array. Hence, we simulated potting a cymbal array with 15 different rubber and epoxy materials available in the market, using the finite element method, and analyzed their effect on the transmitting voltage response spectrum of the array. From the analysis results, we selected the material that would achieve the widest frequency bandwidth, while preserving the structural stability of the array. A potting method corresponding to the selected material was suggested as well. This study provides guidelines for the selection of a potting material for use in underwater transducer arrays.

## 1. Introduction

Since their inception, cymbal transducers have been recognized for their simple structure, low cost, and excellent acoustic performance in underwater communication and detection systems. The array is an important part of a sonar system, but it encounters a harsh marine environment in practical use. Its performance and efficiency can only be guaranteed if its safety is maintained.

To ensure stable operation and avoid the influence of environmental factors, such as moisture, corrosion, and external impacts, the cymbal array can be protected by potting [1,2]. Potting is the process of insulating the array with polymeric materials having a high bonding ability, small expansion coefficient, high mechanical endurance, and strong corrosion resistance. The array and potting materials are made into a solid whole, which is waterproof and resistant to shock and vibration [3]. Because an acoustic transducer array is the object to be potted in this study, the potting should not only ensure protection but also consider the acoustic performance.

The cymbal transducer has been studied and improved upon by many scholars since its invention. Zhang et al. [4,5] designed a cymbal transducer structure that consisted of a piezoelectric-ceramic disk and two metal end-caps that contained a shallow cavity on their inner surface. The metal caps convert and amplify the small radial deformation of the disk into a much larger axial deformation normal to the surface of the caps. Tressler et al. [6,7] continued to redesign the end-cap of the cymbal transducer using different materials and shapes to increase the bandwidth.

Dogan et al. [8,9] used the finite element method (FEM) to study the variation in the transmitting voltage response (TVR), free-field voltage sensitivity, and mechanical quality factor, according to different end-cap and piezoelectric materials. Shim and Roh optimized the structure of a cymbal transducer to achieve a wider bandwidth while maintaining the TVR over a certain level [10]. Howarth et al. [11] conducted a detailed analysis of 7 × 7 and 8 × 8 arrays in an attempt to increase the acoustic output power of a cymbal transducer by increasing the emission area and studied several characteristics of the array.

Although several studies have been conducted on cymbal-array structure designs, as reviewed above, work on insulating the array has not been as active. Tressler et al. [2] attempted two mounting schemes on their cymbal array: unpotted (oil-filled) and potted in a 5 mm-thick stiff polyurethane layer. The two protection treatments resulted in a noticeable difference in the performance of the array. Zhang et al. [12] also used polyurethane to pot cymbal arrays. Rajapan et al. [13] conducted potting tests on cymbal arrays using epoxy and polyurethane.

For underwater acoustic transducers, in addition to offering protection, the potting material must match the acoustic impedance between the array and water. Good acoustic impedance matching results in a wide frequency bandwidth of the array. However, most polymeric materials applicable to potting exhibit certain damping effect on the array. A good potting material will only cause a small decrease in the TVR level of the array. Hence, much care should be taken when selecting the potting material for acoustic transducers.

Furthermore, the cymbal transducer is sensitive to the effect of potting, owing to its inherent nature of operation; that is, the flexural vibration of the thin metallic end-caps. However, very few of the polymeric potting materials available for underwater insulation have been tested in previous studies on cymbal transducers. For both good protection and acoustic performance, a more rigorous approach should be adopted to select the material and method for potting cymbal arrays.

In this study, we aimed to identify a high-performance potting material and an efficient method for potting cymbal arrays. We selected various epoxy and rubber materials known for good insulation and reliability among those available on the market, and used the FEM to simulate potting a cymbal array with them to determine the best material to widen the array bandwidth. A reduction in the TVR level of the array, owing to potting, was inevitable, but we aimed to minimize this reduction. In means that we wanted to increase the gain-bandwidth product of the array by widening the bandwidth. After determining the potting material, we investigated an appropriate method for potting the cymbal array with the material. An experimental cymbal-array specimen was fabricated and potted with the selected material to verify the efficacy of the potting material.

## 2. Finite Element Model of the Cymbal Transducer and Array

**Figure 1:**
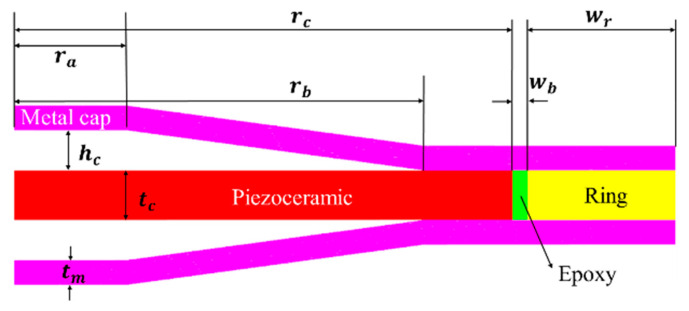
2D axisymmetric finite-element analysis (FEA) model of the cymbal transducer.

This study used PZFlex^®^, a commercial finite element analysis (FEA) software package, to analyze the effect of potting. A cymbal transducer is composed of a piezoceramic disk, a plastic ring, and two caps. The materials of the caps and ring were brass and polyetheretherketone (PEEK), respectively. The PZT-5H piezoceramic was used [14]. In the fabricated specimen, the caps and ring were connected with epoxy (EB-106, EpoxySet, Inc., Woonsocket, RI, USA). However, because the epoxy layer was so thin in the order of several micrometers, the layer was ignored in the simulation model [10]. Figure 1 and Table 1 show schematically the structure and dimensions of the cymbal transducer modeled using the FEM.

**Figure 2:**
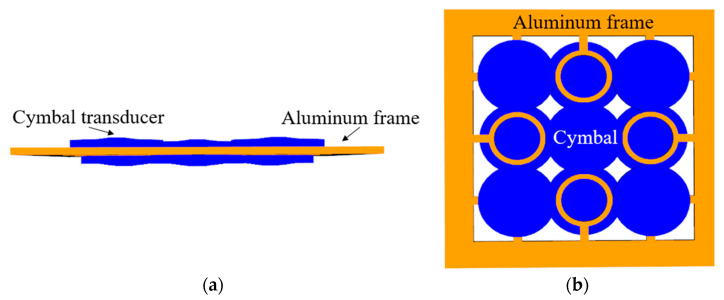
Schematic of the double-layered cymbal array: (**a**) front view; (**b**) top view.

**Figure 3:**
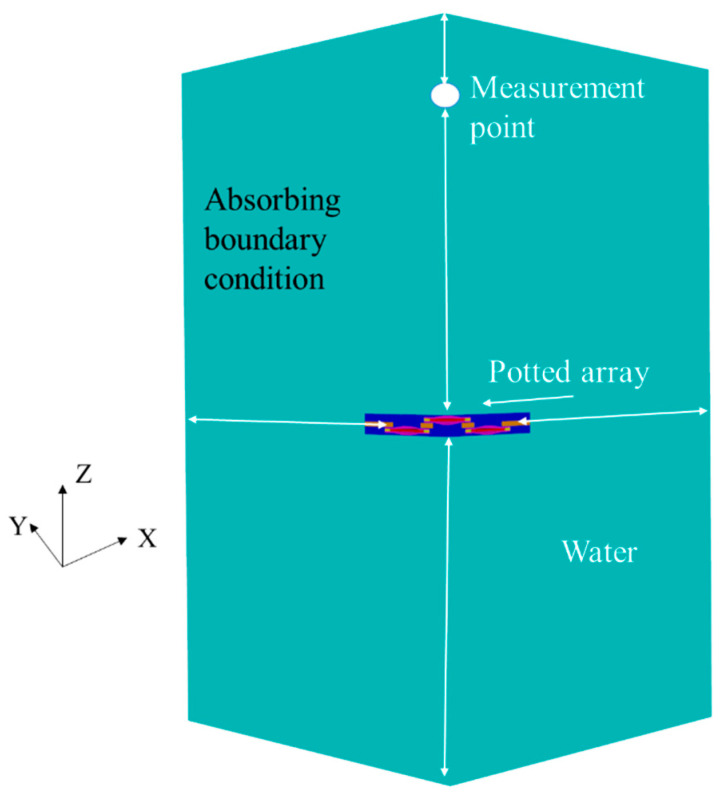
FEA model of the immersed cymbal array.

The array is a double-layered cymbal array, as shown in Figure 2, where the blue area is the cymbal transducer and yellow area is an aluminum frame. The dual-layer array structure could provide a wider bandwidth by reducing the center-to-center spacing between cymbal transducers in the array compared with that of conventional single-layer array structure [15]. The upper and lower layers have five and four cymbals, respectively. The two layers of cymbals are fixed to and supported by an aluminum frame. Figure 3 shows the FEA model of the immersed cymbal array. All transducers in the array were electrically connected in parallel.

Because both the cymbal transducer and array have axisymmetric structures, a 3D quarter model was used to save analysis time. The array was surrounded by sufficient water to preserve the far-field distance from the array to a measurement point around the edge. All the edges of the water domain were enforced with sound-absorbing boundary conditions to prevent any reflection of waves at the boundary.

## 3. Selection of Potting Material

The potting material is positioned between the water and array, so it plays not only a protective role but also an acoustic matching role. To ensure proper array insulation, the potting material should not deteriorate the acoustic performance of the array. The TVR level and −3 dB fractional bandwidth (FBW) are two representative parameters that measure the performance of underwater acoustic transducer arrays. A good potting material should preserve a higher TVR level and wider FBW of the array after potting.

**Figure 4:**
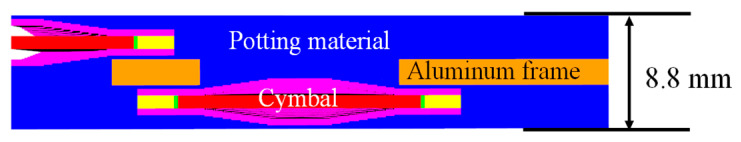
FEA model of the potted cymbal array.

Figure 4 shows a close-up view of the proposed array model. The potting material covers the entire array attached to the aluminum frame. The length, width, and height of the model are 49.2, 49.2, and 8.8 mm, respectively.

For the potting material shown in Figure 4, we tested a variety of representative epoxy and rubber materials available on the market for insulating underwater acoustic transducers. The epoxy materials used were Araldite CY208, CY221, CW1312, HY956, and MY750 (Huntsman, TX, USA); HP20 resin (Mitsubishi Chemical Group, Hsinchu, Taiwan); Silicone Kerf Pemko S104 (Assa Abloy, Stockholm, Sweden); and Stycast W67 (Loctite®, Dusseldorf, Germany). The acoustic properties of the epoxy materials are listed in Table 2.

**Figure 5:**
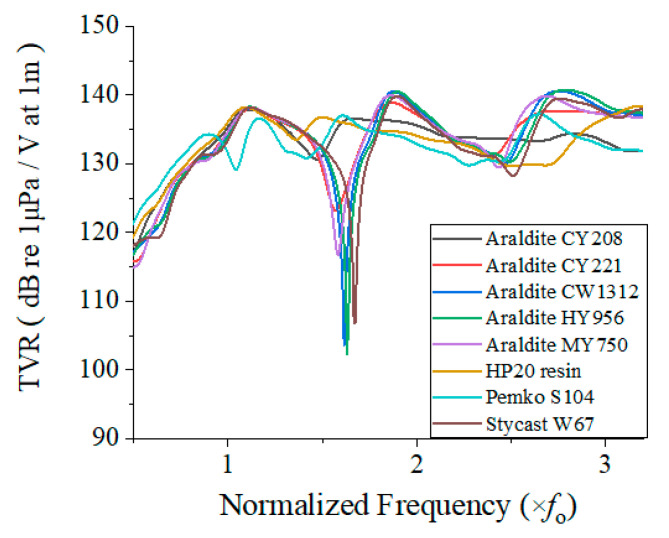
TVR spectra of the array potted with epoxy materials.

Using the model shown in Figure 4, we analyzed the effect of the potting materials on the TVR spectrum of the cymbal array using FEM. In calculating the TVR spectrum, only the potting material was changed; all the other parts of the array remained unchanged. The center frequency of the individual cymbal transducer was *f*_0_, and the frequency scale of the spectrum was normalized to *f*_0_, as shown in Figure 5.

The TVR spectra were significantly different from each other, implying a significant difference in the effect of each material. Five relatively stiffer materials (Araldite CW1312, CY221, HY956, MY750, and Stycast W67) caused clear harmonics of the fundamental mode at *f*_0_, which resulted in a sharp notch at 1.7 *f*_0_. The high stiffness is reflected in the high acoustic impedance of the materials, as presented in Table 2. Furthermore, the lower the shear velocity of the material, the smaller was the fluctuation in the TVR level of the array potted with the material, which led to a wider bandwidth. The quantitative acoustic characteristics were extracted from the TVR spectra and are summarized in Table 3.

According to Table 3, the maximum peak TVR level was observed with Araldite HY956, which reached 140.8 dB, and the minimum was observed with Pemko S104, which was 137.2 dB. In terms of the bandwidth, the performance of the eight materials in the epoxy group was poor. The largest FBW was observed for Araldite CY208 (28.4%). Considering that we need a potting material that preserves a higher TVR level, as well as a larger FBW, the epoxy materials are worse than the rubber materials, as discussed in the next section.

In a manner similar to that for the epoxy materials, the effect of the rubber potting materials on the TVR spectrum of the cymbal array was analyzed using FEM with the model shown in Figure 4. The rubber materials used for potting were Aqualene (Olympus, Tokyo, Japan), Devcon Plastic Steel Liquid (ITW, Glenview, IL, USA), PDMS (Wego Chemical Group, Great Neck, NY, USA), RTV55 (Kem-Tron Inc., Caledonia, MI, USA), RTV664 (Momentive, Waterford, NY, USA), RTV3460 (Elkem, Oslo, Norway), and urethane (Urethane Innovators, New Bern, NC, USA). The acoustic properties of the rubber materials are summarized in Table 4.

**Figure 6:**
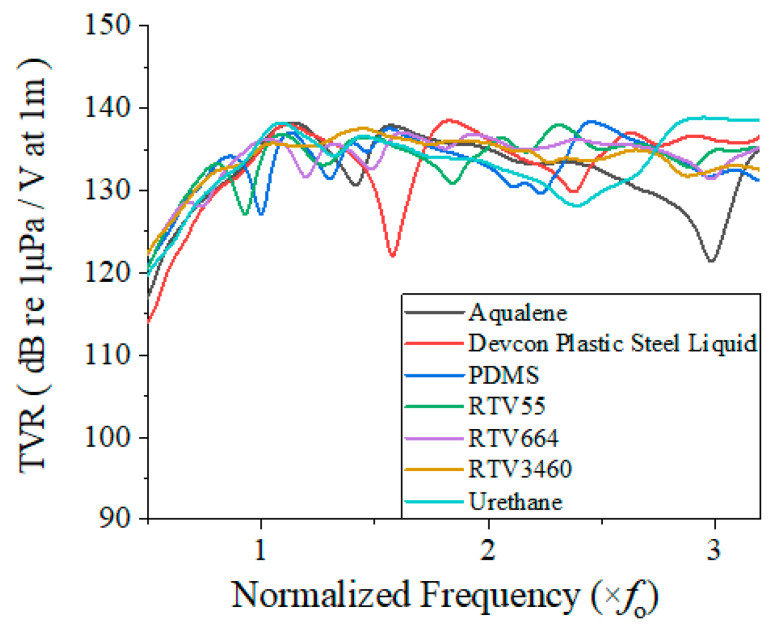
TVR spectra of the array potted with rubber materials.

The results of the analysis are presented in Figure 6, which illustrates the superiority of the rubber materials over the epoxy materials in terms of overall performance. Only Devcon showed a significant drop in the TVR level at approximately 1.7 *f*_0_, whereas other materials showed no considerable downward trend. Aqualene, Devcon, and urethane exhibited relatively large fluctuations. According to the physical properties listed in Table 4, the lower the shear velocity, the smaller the fluctuation in the TVR spectrum.

The quantitative acoustic characteristics were extracted from the TVR spectra and are summarized in Table 5. As with the epoxy materials, a material with an acoustic impedance closer to that of water was more beneficial for the array performance. The highest peak TVR level was observed with Aqualene, reaching 140.1 dB. A higher FBW was observed for materials with lower shear velocities, and the array potted with RTV3460 had the largest FBW of up to 78%. Although the peak TVR level of the array potted with RTV3460 was not the highest, the difference from those of the array potted with other materials was not significant; that is, less than 3 dB.

The purpose of this study was to determine the best potting material to obtain the widest array bandwidth. Hence, of the 15 materials tested, RTV3460 was selected as the final potting material. RTV3460 has been used as an insulating material for many applications [16]. RTV3460 has been also commonly used for the acoustic lenses in medical ultrasound probes [17,18], which proves the endurance of RTV3460. Tear strength, usable temperature range, and shore A hardness of RTV3460 are 25 kN/m, −54 °C to 204 °C, and 58, respectively, while linear shrinkage is less than 0.1% [19,20]. This material is suitable for potting the cymbal array with good electrical isolation and mechanical durability in addition to its acoustical performance.

## 4. Selection of Potting Method

**Figure 7:**
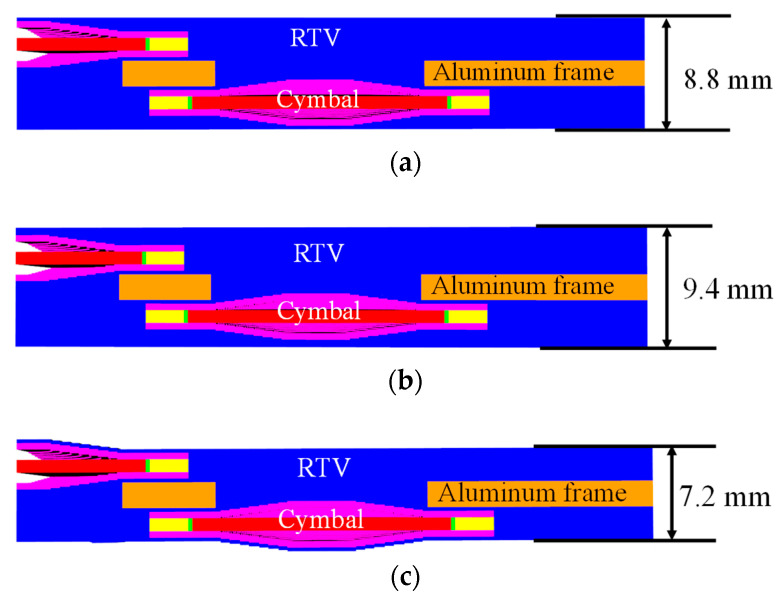
Schematic of the cymbal array potted by: (**a**) method 1; (**b**) method 2; (**c**) method 3.

As the next step, we attempted to find an appropriate method to pot the cymbal array with the polymeric material determined in Section 3. Figure 7 shows the schematic of the potted arrays, and we tried to select a proper potting method by considering the feasibility and ease of fabrication. The potting material used was RTV3460, as described in Section 3. In Figure 4, the overall thickness of the double-layered cymbal array, including the frame, was 8.8 mm.

To better protect the array, we changed the thickness and pot geometry. An increase in the potting-material thickness was likely to have a negative impact on the TVR level of the array, because polymeric materials have a damping effect on sound waves [21]. However, a decrease in the sharp peak in the TVR spectrum may contribute toward widening of the bandwidth of the array.

In Figure 7a, the structure of the array potted using method 1 is the same as that of the original potted structure in Figure 4. The entire potted array had a uniform thickness of 8.8 mm. The structure of the array potted using method 2 in Figure 7b is the same as that in Figure 7a; however, the overall thickness of the potting material was increased by 0.6 mm to 9.4 mm. In our preliminary analysis, an excessive increase in the thickness caused an irrecoverable reduction in TVR level of the array; therefore, the increase was minimized to investigate the effect of a larger thickness.

The array potted by method 3 in Figure 7c has a thin layer of thickness 0.3 mm on the surface of the caps. This potted array has different thicknesses at different points; 7.2 mm is the thickness at the thinnest point of the array. The purpose of this structure is to minimize the loss of radiated acoustic power, while achieving the bandwidth-widening effect of the potting material.

**Figure 8:**
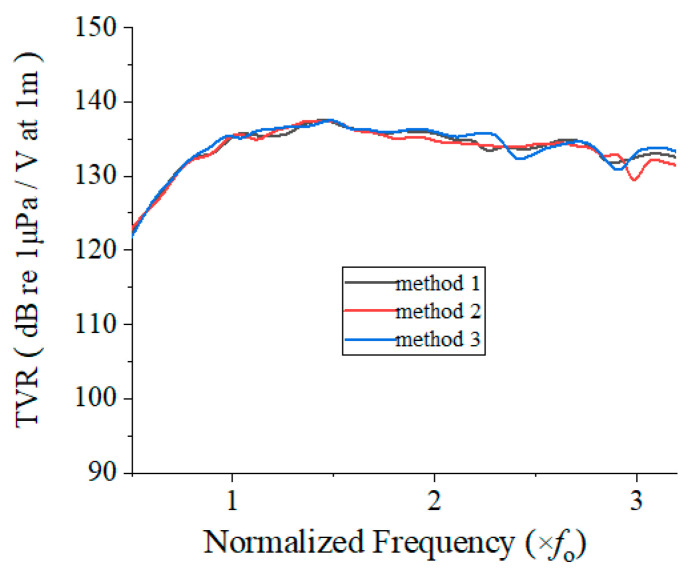
TVR spectra of the array potted by methods 1–3.

The effects of the three potting methods were analyzed using the FEA model shown in Figure 3 by calculating the TVR spectrum of each array. The results are shown in Figure 8. The three TVR spectra were quite similar, implying that there was no noticeable difference in the effect of the three potting methods. Achieving a high peak TVR level and a wide bandwidth in the cymbal array was the goal of this study; however, the premise of the acoustic performance was to ensure a safe and stable operation of the array. Because there was no significant difference in the acoustic effect of the three methods, method 2 was selected as the potting method for the safe protection of the array because of its thicker structure.

## 5. Fabrication of a Potted Array

To verify the rationality of selecting the potting material and method, we fabricated a cymbal array specimen and measured its TVR spectrum. In the previous sections, we chose RTV3460 as the potting material and Potting method 2 as the final potting method. Our fabricated array was a 5 × 5 cymbal array, instead of a 3 × 3 array. We increased the number of cymbal transducers in the array to improve the signal-to-noise ratio in the measurement; however, the structure and material of the fabricated 5 × 5 array were exactly the same as those of the 3 × 3 FEA model shown in Figure 2. Twenty-five cymbals were arranged in double layers, with 13 cymbals in the upper layer and twelve in the lower layer.

**Figure 9:**
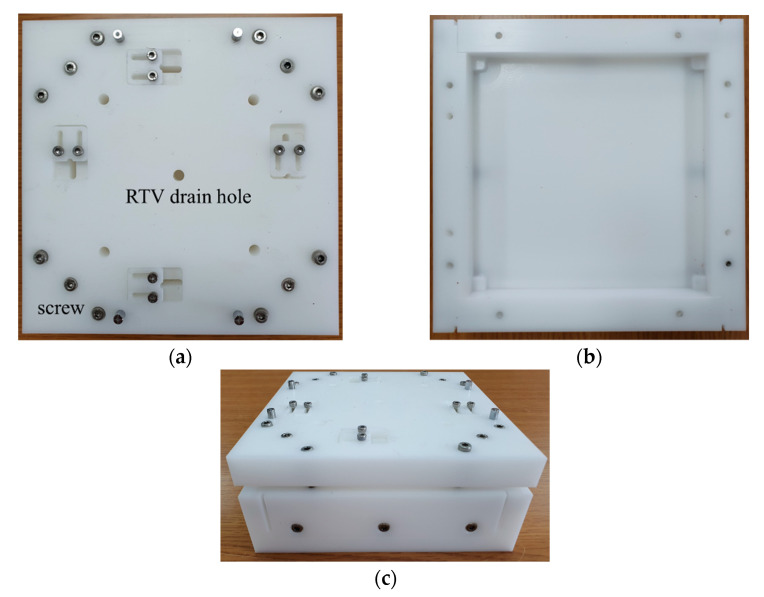
Photograph of the potting mold: (**a**) top plate; (**b**) bottom plate; (**c**) top and bottom plates assembled.

To facilitate the potting process, we made a mold exclusively for the potting. Figure 9 shows the mold made of acetal, which consists of top and bottom plates. The first step to make a potted array was to pour the liquid RTV3460 into the bottom plate. Meanwhile, the cymbal array was bolted to the upper plate. To remove air bubbles observable in the RTV, the bottom plate filled with RTV3460 was placed in a vacuum chamber for 10 min, which was called ‘degassing process’. Next, the upper plate on which the cymbal array was fixed was assembled with the bottom plate steadily using bolts. It is important to note that if the assembly time was too fast, many air bubbles could be generated. Therefore, we spent sufficiently long time in combining the top and bottom plates, i.e., by bringing the top plate closer to the bottom plate by 1 mm per 10 min. The top plate has holes to extract extra RTV3460 that may contain air bubbles. After the top and bottom plates were assembled, the mold assembly was placed in an oven to harden the liquid RTV3460.

**Figure 10:**
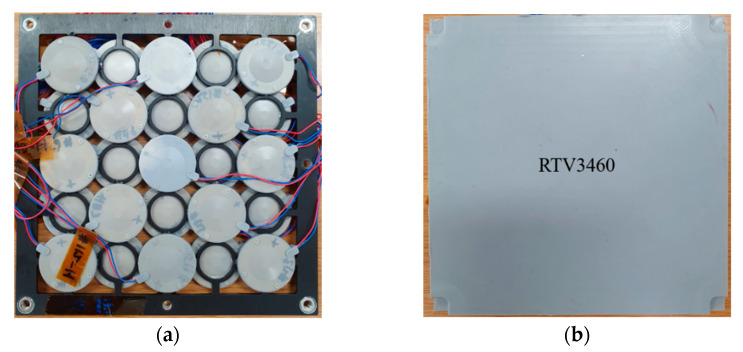
Photograph of the fabricated cymbal array: (**a**) before potting; (**b**) after potting.

Figure 10 shows the array before and after potting. Electrical wires were attached to the cymbal transducers and connected in parallel to a power amplifier.

**Figure 11:**
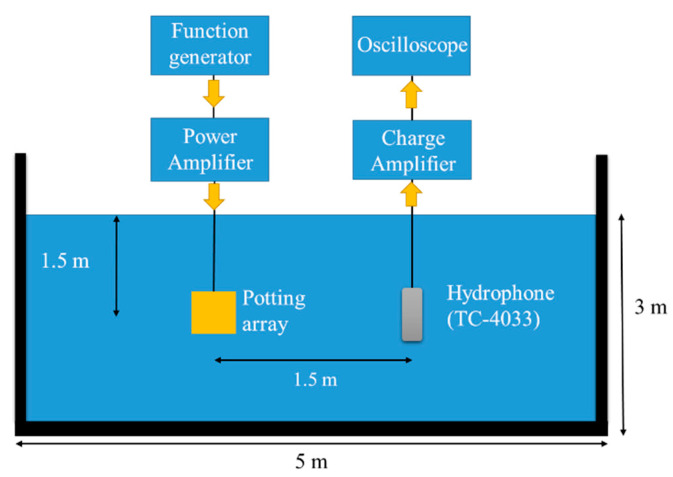
Experimental setup for underwater TVR spectrum measurement.

**Figure 12:**
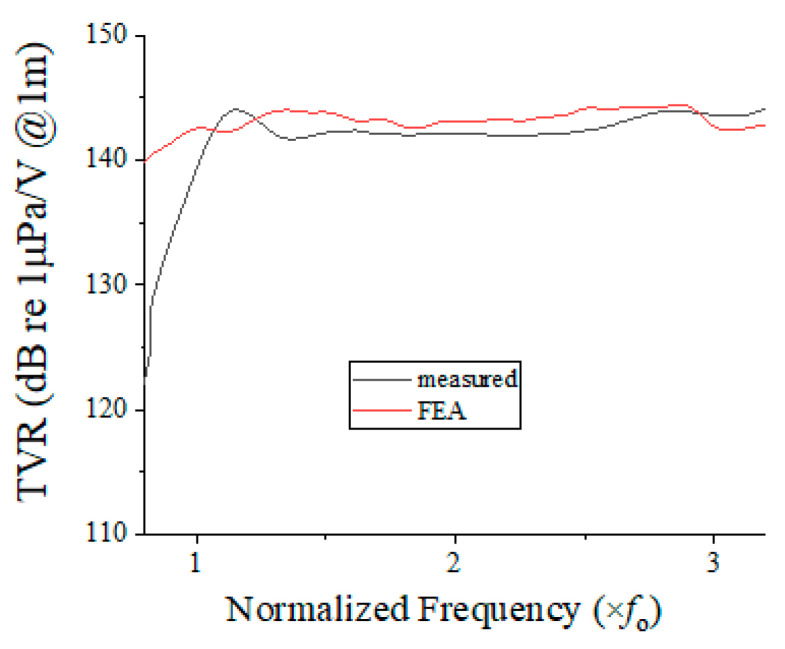
TVR spectra of the potted array.

The underwater TVR spectrum of the fabricated array was measured using the experimental setup illustrated in Figure 11, which is the same facility as was used in [10]. Figure 12 presents the TVR spectrum obtained from the measurement compared with that obtained from the FEA. The measured spectrum was in good agreement with the analyzed spectrum. The −3 dB FBW from the measured spectrum was 101.9%, whereas that from the FEA was 95.8%. The difference between the two FBWs can be attributed to the mechanical tolerances in fabricating the prototype array. The good agreement validates the results of the analyses conducted in Section 3 and Section 4.

## 6. Conclusions

Because a cymbal array is typically used as an underwater projector, it must be protected from the harsh underwater environment while preserving its acoustical properties. Therefore, using PZFlex^®^, we simulated potting of the cymbal array with 15 representative epoxy and rubber materials available on the market for insulating underwater acoustic transducers, and analyzed their effect on the TVR spectrum of the array. The goal of this work was to find a high-performance potting material to obtain the widest array bandwidth.

Among the tested materials, RTV3460 was selected as the potting material, and method 2 in Figure 7 was selected as the corresponding potting method. The effect of the selected potting material and method was validated by fabricating a prototype cymbal array and comparing its measured TVR spectrum with the analyzed spectrum. The comparison showed a good agreement, confirming the validity of the selection. The potting material selected in this study can be applied to protecting general underwater transducers, while maintaining their acoustic characteristics.

## Figures and Tables

**Table 1:** Structural parameters and dimensions of the cymbal transducer.

Structural Parameter	Symbol	Value (mm)
Apex radius of the cap	*r_a_*	2.3
Base radius of the cap	*r_b_*	8.2
Radius of the piezoceramic disk	*r_c_*	10.0
Height of the cavity	*h_c_*	0.8
Thickness of the piezoceramic disk	*t_c_*	1.0
Thickness of the cap	*t_m_*	0.5
Width of the ring	*w_r_*	3.0
Width of the epoxy	*w_b_*	0.3

**Table 2:** Acoustic properties of the epoxy materials.

Potting Material	Acoustic Impedance (MRayl)	Density (kg/m^3^)	Longitudinal Velocity (m/s)	Shear Velocity (m/s)	Longitudinal Attenuation (dB/m)	Shear Attenuation (dB/m)
Araldite CW1312	2.91	1149	2536	1179	2.9	7.4
Araldite CY208	2.32	1165	1989	762	16.3	115.2
Araldite CY221	2.78	1134	2452	1110	9.0	41.1
Araldite HY956	3.05	1146	2658	1237	4.0	12.6
Araldite MY750	2.72	1150	2365	1110	2.5	7.6
HP20 resin	1.80	1122	1605	549	5.7	102.8
Pemko S104	1.38	1243	1114	223	2.0	25.0
Stycast W67	4.72	2007	2350	1139.9	2.7	6.0

**Table 3:** Acoustic characteristics of the array potted with epoxy materials.

Potting Material	Peak TVR (dB)	Bandwidth (×*f*_0_)	Center Frequency (×*f*_0_)	FBW (%)
Araldite CW1312	140.6	0.41	2.82	14.5
Araldite CY208	137.9	0.32	1.16	28.4
Araldite CY221	138.9	0.32	1.92	16.7
Araldite HY956	140.8	0.43	2.86	15.0
Araldite MY750	139.9	0.26	1.92	13.5
HP20 resin	138.2	0.31	1.13	27.4
Pemko S104	137.2	0.27	2.68	10.1
Stycast W67	139.9	0.22	1.93	11.4

**Table 4:** Acoustic properties of the RTV materials.

Potting Material	Acoustic Impedance (MRayl)	Density (kg/m^3^)	Longitudinal Velocity (m/s)	Shear Velocity (m/s)	Longitudinal Attenuation (dB/m)	Shear Attenuation (dB/m)
Aqualene	1.46	921.5	1593	659	2.61	26.9
Devcon Plastic Steel Liquid	3.34	1817	1836	973	7.38	16.0
PDMS	1.08	1050	1030	200	1.48	52.1
RTV55	1.40	1420	987	167	0.17	19.1
RTV664	1.32	1294	1022	125	4.08	17.4
RTV3460	1.18	1198	983	45	1.77	7.1
Urethane	1.59	1020	1560	500	25.0	75.0

**Table 5:** Acoustic characteristics of the array potted with RTV materials.

Potting Material	Peak TVR (dB)	Bandwidth (×*f*_0_)	Center Frequency (×*f*_0_)	FBW (%)
Aqualene	140.1	0.64	3.82	16.8
Devcon Plastic Steel Liquid	138.5	0.32	1.88	17.0
PDMS	138.3	0.35	2.54	13.8
RTV55	137.9	0.56	2.48	22.6
RTV664	137.1	1.30	2.18	59.6
RTV3460	137.5	1.24	1.59	78.0
Urethane	138.2	0.29	1.12	25.9

## Data Availability

Not applicable.
